# The Adoption of Preventive Behaviors during the COVID-19 Pandemic in China and Israel

**DOI:** 10.3390/ijerph17197170

**Published:** 2020-09-30

**Authors:** Xue-Jing Liu, Gustavo S. Mesch

**Affiliations:** 1 School of Public Health, University of Haifa, Haifa 31905, Israel; xliu@campus.haifa.ac.il; 2 Joint Translational Institute of Science and Technology, East China Normal University, Shanghai 200062, China; 3 Department of Sociology, University of Haifa, Haifa 31905, Israel

**Keywords:** COVID19, social distance, preventive behaviors, 3C’s model

## Abstract

The COVID-19 pandemic represents a massive global health crisis. The rapid transmission rate of the virus, as well as the lack of effective medications and vaccines, has posed serious challenges to controlling the spread of the disease. Dealing with this public health crisis has required major changes in people’s behavior, including the adoption of social distancing measures such as avoiding meeting with family members and friends, crowded places, and public transportation. The purpose of this study is to investigate the factors associated with the adoption of these behaviors in China and Israel. We relied on the 3Cs model that has been used to predict the adoption of a specific preventive behavior (vaccinations) with the goal of testing its applicability to other preventive behaviors such as in response to the COVID-19 pandemic. The model indicates that confidence in social institutions, complacency (fear of and assessments about the risk of becoming ill) and constraints (levels of self-efficacy and confidence in being able to engage in the behaviors) are predictors of adopting preventive behaviors. Data were collected in China and Israel through an online survey of the population (*n* = 1406). We used latent variables and structural equation modeling to test the hypotheses derived from the 3Cs model. The findings indicate that there are some differences in the types of preventive behaviors adopted in the two countries. In Israel, higher levels of confidence predicted the adoption of avoidant behaviors and more constraints predicted engaging in fewer avoidant behaviors. In China, more constraints also contributed to the adoption of fewer avoidant behaviors, but people’s level of confidence fully mediated this result. The multi-group analysis indicated that the conceptualized model fits the Chinese and Israeli data reasonably well. The findings suggest that the 3Cs model can be generalized from getting vaccinated to adopting avoidant behaviors and that the model can be used across cultures and countries.

## 1. Introduction

At the end of 2019, the COVID-19 pandemic broke out initially in Wuhan, China, and expanded rapidly, creating a massive public health crisis worldwide. During our data collection period in April 2020 the World Health Organization reported over 1 million confirmed cases globally, resulting in a more than tenfold increase in less than a month [1].

The rapid transmission rate, and the lack of available and effective pharmaceutical treatments and vaccines, required the use of non-pharmaceutical interventions. Examples included social distancing behaviors such as using masks, keeping spatial distance, avoiding crowded places and meeting with friends and family members. These behaviors were believed to be effective in reducing the spread of the disease [2]. Many national authorities and international health organizations such as the Israeli Ministry of Health, the National Health Commission of the People’s Republic of China and the World Health Organization published guidelines to provide individuals with recommended behaviors. National governments enforced these guidelines based on local situations.

The adoption of preventive behaviors depends on the willingness of the public to engage in radical behavioral changes. Since these behavioral changes involve significant social and economic costs [3,4], both governments and individuals have to balance the cost and benefits of modifying their daily activities. Therefore, personal, social, cultural, and developmental factors affect the decision to adopt these behaviors [5,6].

Israel and China are at different points on the epidemiological curve. Israel reported its first confirmed imported case on 21 February 2020, about two months later than the suspected cases in Wuhan, China [7]. On April 1, 2020, China reported 82,631 cumulative confirmed cases, and Israel reported 5129. China had 3321 deaths, 157 times higher than Israel. During this period the two countries differed in their adoption of avoidant behaviors.

From the perspective of a society, behavioral changes as a response to public health recommendations are heavily dependent on the acquisition of new social norms of interaction. Social contact patterns and hygiene behaviors are habitual and are difficult to modify. The COVID-19 pandemic provides us with a natural laboratory for the investigation of rapid and dramatic changes in social behavior [8].

As a country with a Western culture, Israelis are more likely than the Chinese to express their emotions with close physical contact such as kissing and hugging [9]. The behaviors involved in social distancing are antithetical to Israeli society. In China, the COVID-19 pandemic coincided with the Chinese spring festival, during which it is customary to participate in family reunions and visit friends and family. Imposing social distancing demands during this period was very challenging for Chinese officials. As to cultural differences [10], Israeli society is more inclined to individualism, whereby personal desires often outweigh social demands. In contrast, China is a typical collectivistic society characterized by a strong commitment to social norms and strict punishment for deviation from these norms [11,12,13].

Given these similarities and differences, we can obtain useful insights into the factors that affected how people regarded the outbreak, the authorities, and their surroundings, and their subsequent adoption of avoidant behaviors or failure to do so. To accomplish this goal, we utilize the 3Cs model that was created to determine whether or not people would get vaccinated. The 3C’s model was originally used as a framework to analyze the three major components of the vaccine hesitancy in the Strategic Advisory Group of Experts (SAGE) on Immunization, a multidisciplinary working group of scholars and practitioners with the World Health Organization in 2012 [14]. Since them, this model has been used in various health programs [15,16] to understand how individuals’ confidence, complacency, and constraint influence the complex decision-making process on immunization [17]. We assume that vaccines are a special case of preventive behavior. In the absence of any vaccine for the COVID-19 virus, we should investigate whether the factors involved in the 3Cs model--confidence, complacency and constraint—can be used to determine whether or not people will adopt the preventive behaviors recommended by public health authorities such as social distancing. Furthermore, we explore the ability to generalize the findings to China and Israel, two countries that vary a great deal in the nature of their societies. Doing so will provide evidence to evaluate the extent to which the 3Cs model is universal and invariant according to culture

## 2. Literature Review

### 2.1. Confidence

Confidence plays a central role in the effort to establish and develop the legitimacy of public health actions, coordinate complex relationships in the health system and govern human behaviors [18,19]. Confidence in this context has been defined as expectations about the ability, reliability and competence of leading social institutions, the healthcare system, their professionals and policy makers to perform satisfactorily during a public health crisis [20,21,22]. Confidence in key social institutions is a significant predeterminant of compliance with recommended behavior [23].

Trust in the micro- and macro-levels of social institutions is inter-connected [24]. Government plays a key role in policy-making and communication during a crisis. In such situations, the public assesses its competency in dealing with the crisis in the absence of a vaccine and expects a transparent information strategy. The public also expects hospitals to be able to treat the seriously ill, isolate the mildly infected and test suspicious cases. Finally, the public must also trust medical personnel [24] as experts whose actions reaffirm the system’s trustworthiness [25].

Previous studies have shown that levels of trust in social institutions are associated with greater willingness to comply with preventive recommended behaviors during SARS, Influenza A/H1N1 [26,27], the Ebola outbreak [28,29], and COVID-19 [30]. Nevertheless, people’s confidence in institutions is dynamic and flexible. They respond to sensational events and their accompanying impact [31] with suspicion. Conspiracy theories are often widespread and may challenge their confidence in social institutions [32,33,34]. When people’s trust erodes, they may be less likely to engage in prescribed changes in their behavior [35].

Thus, we expect a positive association between confidence in social institutions and the adoption of avoidant behavior among Israeli and Chinese participants.

### 2.2. Complacency

Complacency is about both the probability of being harmed and the nature of the harmful consequences [36]. With regard to illness, complacency involves assessments about two components: the probability of becoming ill and the severity of the negative outcomes due to illness [37]. When people feel that they face little risk of getting sick, they are unlikely to engage in preventive health behaviors.

The concept of complacency has its roots in the perspective of expected utility. The consequentialist perspective from the expected utility theory emphasizes that the motivation for behavior is based on an assessment of the consequences of possible alternatives that one can choose [38]. Personal behavior can be predicted by assessing the likelihood and severity of the outcome. Based on Tversky and Kahneman’s subjective probability [39], risk perceptions can sometimes be understood as a function of heuristics [40]. The availability heuristic says that people make their assessments about risks based on recent information and the situation. Thus, investigating perceptions about risk is necessary for dealing with certain biasing factors such as constraints and deficiencies [41].

Studies have posited that risk perceptions affect people’s adoption of preventive behaviors. This hypothesis has been tested using a meta-analysis suggesting that people’s susceptibility to a disease and its severity are core indicators of whether people will get vaccinated against the disease [42]. Complacent individuals who feel that the risks are minimal are less likely to take preventive measures [43,44,45]. One empirical study found that complacency is negatively related to the adoption of behavior to protect one’s health [46]. In other words, people who feel they are more likely to become ill are more likely to engage in protective behaviors.

However, some studies have indicated that when the public feels that policy makers and the media are exaggerating the risks, the opposite outcome occurs. As several studies of Influenza A/H1N1 and Ebola have demonstrated, in such cases complacency can also have a negative relationship with the adoption of recommended behaviors.

The relationship between complacency and adopting preventive behaviors during the COVID-19 pandemic is still unclear. Therefore, our goal is to identify the level of complacency of our Israeli and Chinese participants and determine whether and how it influences their adoption of protective behavior.

### 2.3. Constraints

The second construct in the 3C model is constraints, meaning the structural and psychological barriers impeding the adoption of avoidant behaviors [47,48]. Constraints are associated with people’s assessments about their ability to exercise behavioral control [49], self-efficacy in adopting avoidant and protective behaviors [50], and empowerment [51]. It is based on outcome expectations and efficacy expectations [52]. Outcome expectations refer to people’s assessments that adopting the recommended avoidant behaviors will lead to positive outcomes. Efficacy expectations are defined as the conviction that one can successfully engage in the behaviors needed to produce the positive outcome. According to social cognitive theory [53], the combination of these two expectations determines the adoption and persistence of behaviors.

Outcome expectations indicate perceived benefits, defined as beliefs about the beneficial outcomes accrued from actions. It is a leading predictor in the health belief model for health behavior [54]. The transtheoretical model, protection motivation theory, theory of reasoned action and theory of planned behavior have also taken outcome expectations into account when predicting the adoption of a recommended behavior [55,56,57].

Self-efficacy is the belief that one can successfully complete the behavior of interest despite barriers to doing so. It also refers to the behavioral skills needed to do so [58]. Self-efficacy is a source of empowerment, that supports the use of individual strengths and competencies, and proactive behaviors that afford the adoption of avoidant behaviors [59]. Hence, people who do not perceive the empowered outcome of their activities could face constraints to gain control over their lives [60].

Constraints resulting from either outcome expectations or assessments of one’s self-efficacy are critical for compliance with avoidant behaviors in a pandemic. In the case of COVID-19, people who believe they are capable of engaging in the avoidant behaviors but are not convinced that behavioral changes could achieve the desired effects might not adopt the recommendations. Similarly, people who believe that they themselves will determine that outcome, but lack the requisite skills or resources will fail to engage in preventive behavior.

There is theoretical and empirical evidence demonstrating the value of constraints in predicting the adoption of recommended behaviors in pandemics. For example, during the COVID-19 outbreak, people who had higher outcome expectations about engaging in avoidant behaviors were more likely to follow the recommended guidelines [61]. Assessments about self-efficacy also positively impacted intentions to self-isolate during COVID-19, and influenced Americans’ avoidance of domestic travel during the Ebola outbreak [62,63]. During the Influenza A (H1N1) crisis, high levels of assessments about one’s self-efficacy were associated with more statements from participants that they would take avoidance measures [64].

In addition to its direct effect on behavior, constraints can operate indirectly [65]. Bandura proposed that all changes might be mediated by assessments about self-efficacy [66]. Thus, constraints will mediate the relationships between other predicators and compliance with avoidant behavior. This mechanism was discussed during the Influenza A (H1N1) pandemic. Perceptions about the outcome expectations of avoiding crowded places were closely correlated with perceptions about risk [67]. In addition, higher levels of expectations about efficacy were strongly associated with confidence in governments [68]. 

The mediation mechanism deserves more elaborated investigation, especially during pandemics. Therefore, we posited that:

**Hypothesis** **1** **(H1).** *Greater confidence in the ability of social institutions to deal with the outbreak will be positively associated with the adoption of avoidant behavior.*

**Hypothesis** **2** **(H2).** *Higher levels of constraints will be negatively associated with the adoption of avoidant behavior.*

**Hypothesis** **3** **(H3).** *Higher levels of complacency will be negatively associated with the adoption of avoidant behavior.*

**Hypothesis** **4a** **(H4a).** *Confidence will have an indirect effect on the adoption of avoidant behavior through constraints.*

**Hypothesis** **4b** **(H4b).** *Complacency will have an indirect effect on the adoption of avoidant behavior through constraints.*

We tested these hypotheses using information from Israeli and Chinese participants.

## 3. Methodology

### 3.1. Data Collection

We collected the data for this study through online surveys. In each country, the principal investigators contacted well-known commercial companies that have large panels of samples of Internet users and have experience in conducting online surveys. The surveys were conducted at the end of March 2020. Both principal investigators constructed the survey originally in English. The questionnaire had 68 items, and included questions about perceptions regarding the risk of contracting COVID-19, self-efficacy, media use, adoption of avoidant and protective behaviors, and socio-demographic information. The sample size in China and Israel was the same—703 respondents in each country. The final sample included 1406 participants who completed the survey and consented to our using it.

Table 1 presents the characteristics of the sample. In terms of gender, 694 were men and 712 were women. Half of them came from Israel and the other half of them came from China. Most of the participants (93.95%) were in the age groups of 18–30, 31–50, and 51–70, accounting for 39.12%, 36.84%, and 17.99% of the sample, respectively. About 90% of them had completed at least high school or the equivalent. More than half of the survey respondents—53.06%—were married, and 39% were unmarried. About 76% of the participants reported earning an average or below average income according to the national standard in their country.

### 3.2. The Study’s Variables

#### 3.2.1. Confidence in Social Institutions

We measured the participants’ level of trust using three items that asked them to indicate their trust in the ability of the government, hospitals, and medical personnel to deal with the outbreak. Responses to the three items were on a 4-point Likert scale (1 = not at all to 4 = very confident). The three variables were subjected to an exploratory factor analysis and resulted in one factor. For that reason, we created a latent variable of confidence in social institutions to use in the analysis. The latent variable demonstrated good reliability (in China, α = 0.88, in Israel α = 0.85, in both countries α = 0.91). Higher scores indicate greater confidence in the ability of social institutions to deal with the pandemic.

#### 3.2.2. Complacency

We measured the participants’ complacency with four items assessing their perceptions about their susceptibility to the disease and the seriousness of becoming infected. We asked the participants to indicate the degree to which they agreed with four statements on a 5-point scale ranging from 1 (strongly agree) to 5 (strongly disagree). A higher score indicated a higher level of complacency. An exploratory factor analysis determined that all of the variables belonged to the same dimension. Therefore, we constructed the latent variable of complacency using the four indicators. The internal validity of the latent variable was acceptable (in China, α = 0.77, in Israel α = 0.68, in both countries α = 0.67).

#### 3.2.3. Constraints

To measure the participants’ constraints, we asked them to indicate the degree to which they agreed with four statements on a 5-point scale ranging from 1 (strongly agree) to 5 (strongly disagree). A higher score indicated more personal constraints. The items dealt with perceptions about the extent to which they believed that healthy behavior could help protect them from contracting COVID-19 and their confidence in their ability to engage in avoidant behaviors. The scale had an acceptable internal reliability (in China, α = 0.707, in Israel α = 0.55, in both countries α = 0.65). Therefore, we constructed the latent variable of personal constraints with the four variables.

#### 3.2.4. Avoidant Behavior

We measured how frequently the participants engage in specific avoidant behaviors using nine indicators of avoidant behaviors and measured on a 5-point scale range from 1 (never engage in this behavior) to 5 (always engage in this behavior). The items included (1) avoid going to places where it is known that the virus is locally transmitted, (2) do not share your towel with others, (3) maintain at least a 1-m distance between yourself and anyone who is coughing or sneezing, (4) avoid shaking hands, (5) avoid using common utensils in the same meal, (6) do not visit family members and friends who do not live with you, (7) do not go into crowded or unventilated places, (8) put on a facial mask when you go outside, (9) do not use public transportation. An exploratory factor analysis was conducted and resulted in a single dimension. The internal reliability of the scale was Cronbach’s α = 0.729 for both countries combined, 0.764 for Israel, 0.746 for China.

### 3.3. Data Analysis

We used IBM SPSS Statistics for Windows, version 20 (IBM Corp., Armonk, N.Y., USA)) to conduct a descriptive analysis for the variables and a bi-variable correlation analysis between the observed exogenous variables. To examine the relationships in the hypotheses, all of the latent variables were incorporated into a structural equation model (SEM) and fitted with the SPSS Amos 23.0 package. In order to obtain least-biased parameter estimates [69], we used a maximum likelihood estimation to estimate the coefficients of the parameters and test the significance of each path. To assess our assumptions, we included direct paths from the latent variables of confidence, constraints, and complacency to avoidant behavior. We also studied the indirect effect of the latent variable of constraints at the same time. We used standard estimation [(Xi-Xmean)/SD] to calculate the intensity of the direct and indirect effects, which indicated the expected amount of change in engaging in avoidant behavior or the mediator produced by a one-unit change in the corresponding latent variable. After this step, we applied the multi-group analysis technique to test the invariance of the participants in the two countries. For each model operation, we used multiple, established criteria for evaluating the goodness of fit of the model. Specifically, we defined a model as having a good fit with the data when the root mean square error approximation (RMSEA) ≤ 0.06, the goodness-of-fix index (GFI) ≥ 0.90, and the standardized root mean square residual (SRMR) ≤ 0.06.

## 4. Results

Table 2, Table 3 and Table 4 list the variances, covariances, correlations of indicators, and Cronbach’s α for each constructed latent variable. The correlations among the indicators constructed as one latent variable were stronger than the correlations with indicators outside their latent variables. This result supported the evidence that the indicators used to construct the three latent variables did not closely correlate with one another.

The Israeli participants and Chinese participants differed in their levels of the latent variables of complacency. While during the outbreak and in daily life both groups indicated that they thought the virus was serious and they were very susceptible to it, the Israelis thought they were more susceptible to it than the Chinese both in daily life and during the outbreak period. In contrast, the Chinese participants regarded the virus as more serious than the Israelis, both in daily life and during the outbreak period.

For the latent variables of constraints, both the Israeli and Chinese participants thought that engaging in healthy behaviors was beneficial for preventing disease in their daily life. The level of this belief was slightly more intense when compared to the statistics during the outbreak. At the same time, they reported strong assessments about their self-efficacy in being able to engage in healthy behaviors in their daily life or during the outbreak. Nevertheless, the Chinese participants expressed more self-efficacy in this regard than the Israeli participants.

For the latent variable of confidence in social institutions for dealing with the outbreak, both the Israeli and Chinese participants expressed consistent and high levels of confidence in the various institutions. However, the Chinese participants were more confident in the Chinese institutions than the Israeli participants were in the Israeli institutions.

Table 5 presents the descriptive analysis of avoidant behavior.

For avoidant behavior as a dependent variable, the most salient difference between the participants from the two countries was using a face mask when going out. Chinese participants were significantly more likely to adopt this behavior than Israelis (M = 4.69, SD = 0.731 for Chinese participants, M = 2.57, SD = 1.503 for Israeli participants). On average, the Chinese participants were more likely not to share a towel with others and to avoid going to places where it was known that the virus was locally transmitted. However, on average, the Israelis were more likely than the Chinese to avoid public transportation and crowded places.

### Multivariate Analysis

**Figure 1:**
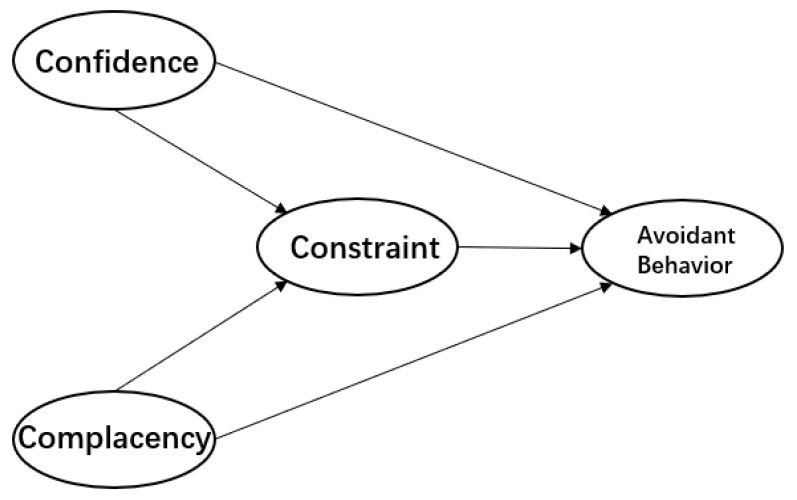
The Conceptualized 3C-avoidant behavior model.

Based on the structural equations and statistical assumptions discussed above, we developed the path diagram of the conceptualized 3Cs avoidant behavior model illustrated in Figure 1.

As Table 6 indicates, the original proposed model was a good fit with the data for the Israeli and Chinese participants. For the Israeli participants, χ^2^(165, *N* = 703) = 646 (*p* = 0.00), χ^2^/df = 3.92, RMSEA = 0.06(0.059–0.070 90% Confidence Interval (CI); GFI = 0.91, SRMR = 0.059. For the Chinese participants, χ^2^(166, *N* = 0 703) = 629 (*p* = 0.00), χ^2^/df = 3.79, RMSEA = 0.06(0.058–0.068 90%CI); GFI = 0.91, SRMR = 0.064.

We used a multi-group analysis to compare the fit of the structural equation models for the Israeli and Chinese participants, and to test the invariance of the model. The unconstructed model showed a better fit [χ^2^(333, *N* = 703) = 1256.452 (*p* = 0.00), χ^2^/df = 3.773, RMSEA = 0.044(0.042–0.047 90%CI); GFI = 0.912, SRMR = 0.06] than other models with invariance constraints (Table 6). The estimations of the standardized coefficients for Israeli participants in the multi-group analysis were identical to the values we found in the previous single model. The same outcome was evident for the Chinese participants. We found no difference in the paths. Therefore, we can conclude that the conceptualized model fit the Israeli and Chinese data reasonably well, even though the parameters for the Israeli and Chinese participants were not equal.

## 5. Discussion

**Figure 2:**
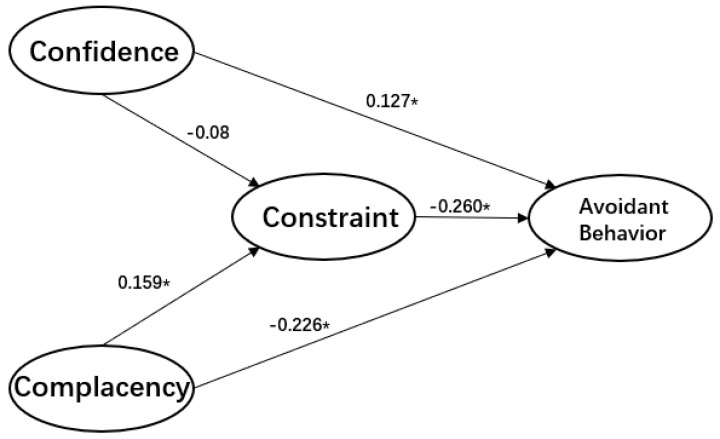
Model estimation of the 3C-avoidant behavior model (Israel), * *p* < 0.05.

**Figure 3:**
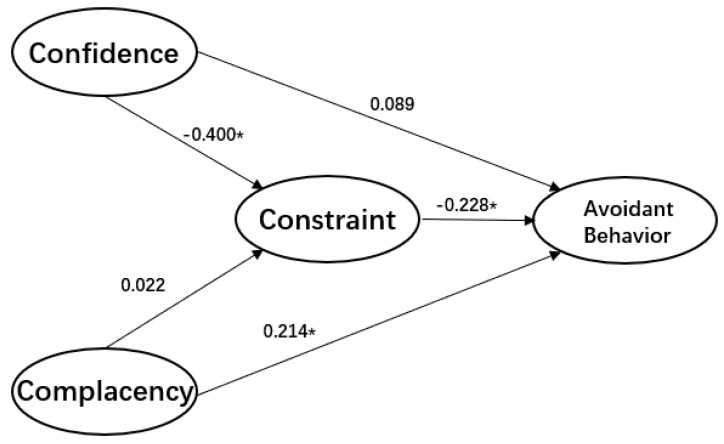
The estimations of conceptualized 3C-avoidant behavior model (China), * *p* < 0.05.

Can the 3Cs model that was developed to determine whether people would get vaccinated to protect their health be applied more widely to determine whether people will engage in behaviors designed to protect them from COVID-19? Furthermore, if we establish that the 3Cs model is useful in this regard, is it applicable to cultures that vary from one another? To answer these questions, we gathered data from Israel and China to investigate the roles of confidence in social institutions, complacency in the face of the threat, and constraints in expectations about the outcomes of engaging in avoidant behavior (see Figure 2 and Figure 3).

The results show that constraints are negatively related to the adoption of avoidant behavior, whereas confidence is positively related to engaging in such behavior. However, these relationships are direct in Israel and indirect in China. In Israel, complacency is negatively related to the adoption of avoidant behavior, whereas in China, this relationship is positive. Therefore, we conclude that the 3Cs model works reasonably well in both the Israeli and Chinese samples, and could be applied to a pandemic.

We found support for our hypothesis that constraints are negatively associated with compliance with avoidant behavior. Indeed, the measurements of it in accounting for the direct effects on both the Israeli and Chinese participants’ behavior were statistically close. This robust relationship contributes to the literature on the role of constraints in complying with healthy behaviors and their indicators. Furthermore, the role of expectations about outcomes and efficacy is becoming increasingly recognized as important antecedents to the adoption and maintenance of recommended behaviors.

At the same time, we found that constraints fully mediated the association between confidence and compliance with avoidant behavior in the Israeli model, and partially mediated this association in the Chinese model. This finding demonstrates the influential role of constraints in behavioral changes. Therefore, we conclude that eliminating perceptions about constraints leading to low expectations about outcomes and efficacy is essential for public health programs and communications, especially during the ever-changing, uncertain period of a pandemic.

Another contribution of this research is identifying the central role of confidence in social institutions as contributing to increased adherence to avoidant behavior, both directly and indirectly. In the Chinese model, the association between confidence and engaging in avoidant behavior was mediated by constraints with an effect of β = −0.40, which extended the differential impact of the hypothesis. This result indicates that confidence in social institutions during the COVID-19 pandemic does not affect the adoption of behavioral changes directly. However, it can remove constraints on expectations and, in turn, increase the likelihood that people will adopt avoidant behavior.

Cultural differences may account for the difference in the direct and indirect effects. For example, the Chinese participants reported much more confidence in social institutions than the Israeli participants. While studies have documented that confidence in the authorities and their executive departments is essential for promoting regulations dealing with public health, it alone does not necessarily ensure that people will engage in such behavior. According to our findings, only when people believe that the behaviors are beneficial (outcome expectations) and they are confident that they can engage in them (efficacy expectations) will they actually adopt them. This finding is consistent with previous research on other pandemics such as Influenza A (H1N1) in Hong Kong, In that study, trust in social institutions was not directly associated with avoidant behavior, but was more strongly associated with efficacy expectations. Thus, efforts should be made to promote more confidence in people’s ability to engage in behavioral change by enhancing their understanding of the outcomes of doing so and developing their efficacy to engage in such behavior. This suggestion highlights the communication dynamics between social institutions, especially governments, and the public. Communication strategies during a pandemic should put more emphasis on encouraging people to engage in avoidant behavior by raising their expectations about the beneficial outcomes of doing so and inspiring their efficacy expectations.

Another interesting finding was that the level of confidence in the government, hospitals, and medical workers was rather close in both Israel and China. Given that the levels of trust in these three groups were similar, we can conclude that the public regards them as sharing responsibility during the pandemic. Therefore, if one party in this triumvirate loses the public’s confidence, the other two remaining parties will be adversely influenced by this loss of trust.

The level of complacency in Israel and China had opposite associations with the adoption of avoidant behavior, and the absolute magnitude of the two associations was close. These divergences could be attributed to the difference in perceptions of complacency between these two groups. The Chinese participants felt they were less likely to become infected than the Israelis. However, if they were infected, they expected the impact to be more severe than the Israelis did.

During the data collection period, the outbreak was gradually brought under control in China. Wuhan emerged from lockdown on April 8, 2020, indicating that the threat of infection had declined. This information might have made the Chinese participants feel that it was less likely that they would become infected. Their stronger sense about the severity of the infection might be due to the large number of deaths and infected people who were still in treatment. In contrast, in Israel, new confirmed cases surged at the end of March, but there were only 20 deaths. Given these statistics, the Israelis might have concluded that they were more likely to become infected, but doing so would not necessarily be fatal.

The combination of the Chinese participants’ perceptions about their relative lack of risk of contracting the virus and the high death rate associated with the disease complicated the role of complacency in the adoption of avoidant behaviors. This positive association between complacency and engaging in avoidant behaviors was consistent with a previous study about Influenza A (H1N1) in which the relatively mild effects of the flu provided little motivation for people to change their behavior [70]. As such, future research should conduct a thorough investigation of the role of complacency in a pandemic.

Another important contribution of this study is our demonstration of the ability to generalize the 3Cs model to contexts other than just becoming vaccinated. Our study is the first to explore the validity of the model in other contexts, particularly with regard to preventive behavior in a pandemic. Despite the fact that the implications of complacency, confidence, and constraints are not identical with regard to being vaccinated and engaging in avoidant behaviors, the model can still provide a mechanism for understanding behavioral change. In accordance with our hypotheses, we indicated that complacency about the possible risks, confidence in social institutions, and constraints regarding expectations are associated with people’s behavior during a pandemic.

Our comparative study also demonstrated the ability to generalize the 3Cs model. We used a conceptual model as a comparative framework to understand how multiple variables function differently in both countries. The Israeli and Chinese participants varied significantly in their levels of complacency, confidence, and constraints, as well as the extent of their engagement in avoidant behavior. For example, both the Israeli and Chinese participants reported significant awareness of the benefits of social distancing. However, the Chinese participants were more optimistic about the situation. They indicated that they felt less susceptible to the disease, had fewer constraints and more confidence than the Israeli participants, but regarded the virus as more serious. Therefore, they were more likely to engage in six out of the nine recommended behaviors. The only exceptions were avoiding shaking hands, crowded, airless places, and public transportation.

Our findings also have several practical implications. First, they suggest that efficient public health campaigns and communications during the outbreak designed to promote the adoption of avoidant behavior must target people’s expectations, particularly with regard to the beneficial outcomes and efficacy of doing so. The goal is to reduce the public’s perceptions about the constraints on their adopting such behavior. Furthermore, these campaigns should promote confidence in social institutions, specifically, the government, hospitals and medical personnel. At the same time, the public’s level of complacency may vary throughout this dynamic situation and deserves a more detailed investigation. Last but not least, the government should work closely with the health care system to build a transparent and responsive communication to the public, to assess individual’s perception, emotional status and increase public support. In the meantime, difficulties in employment, need for daily supplies, epidemic prevention and control supplies, transportation could be the sources of public negative perception and behavior adoption. Policies concerning these issues should be implemented to ease this hardship.

## 6. Limitations

There are some limitations to this study. First, even though the data came from two countries dealing with COVID-19, the data were cross sectional. Our assessments of perceptions and engagement in avoidant behavior were post hoc and cross-sectional. Thus, we could not evaluate causality properly. The cross-sectional nature of these data limits our ability to draw conclusions about causal directions.

Second, although there is extensive research on whether cognition precedes or follows appraisals, there is no consensus on the issue [71]. Previous studies showed various possibilities that might contribute to a particular behavior [72]. In the Israeli model, confidence, complacency, and constraints are antecedents of avoidant behavior, as are complacency and constraints in the Chinese model. Some variables could also influence the behaviors of interest in a serial chain [73].

At the same time, reverse causality might have affected our interpretation of the perceptions associated with avoidant behavior. Engaging in such behavior might promote positive and negative changes in complacency, confidence, and constraints. Social distancing challenges people’s instincts of connecting with others [74]. The isolation and loneliness that stems from social distancing might increase psychological and emotional distress. Sudden changes in people’s social interactions may prove burdensome. As a result, behaviors designed to keep people from contracting the virus might have a deleterious effect on other aspects of their physical and mental well-being, including their cognitive perceptions. This possibility suggests that the association between cognitive perceptions and preventive behaviors is more complex and would benefit from longitudinal studies in the future.

In addition, cognitive perceptions and patterns of social contact are culturally based. They are rooted in social norms of behavior, and culturally specific socialization patterns. In this research, we did not collect data about indicators of cultural values. Meanwhile, differences in lifestyle and daily routine could impact individuals’ decision-making processes during the pandemic, and therefore could change the dynamic of virus transmission. These differences roused by geographic, institutional, and social factors could complex the cross-cultural comparison research that has not been included in this study. Doing so would help us determine the interaction between cultural factors and people’s perceptions and behaviors, promoting international comparisons.

Third, the study may suffer from some potential bias. The use of an online survey meant that we could reach only participants who were Internet users. These participants are more likely to receive instant information about the pandemic and may be more avid followers of the news. These factors could bias the results. Furthermore, the data were self-reports, leading to the possibility of social desirability bias resulting from pressure to conform to social expectations. Nevertheless, despite these limitations, our study benefits from the fact that we asked about the actual behaviors people had adopted, not their future plans for doing so. We were also able to validate the possibility of applying the 3Cs model to dealing with the COVID-19 pandemic and provided a cross-national comparison of our hypotheses.

## Figures and Tables

**Table 1:** Characteristics of sample composition.

Demographic Variable	Both	Israel	China
Sample (%)	1406 (100%)	703 (50%)	703 (50%)
Gender			
Male	694 (49.36%)	336 (23.90%)	358 (25.46%)
Female	712 (50.64%)	367 (26.10%)	345 (24.54%)
Age			
Under 17 years old	36 (2.56%)	0	36 (2.56%)
18–30 years old	550 (39.12%)	194 (13.8%)	356 (25.32%)
31–50 years old	518 (36.84%)	262 (18.63%)	256 (18.21%)
51–70 years old	253 (17.99%)	199 (14.15%)	54 (3.84%)
Older than 70 years old	49 (3.49%)	48 (3.41%)	1 (0.07%)
Education			
No schooling completed	28 (1.99%)	16 (1.14%)	12 (0.85%)
Primary school	21 (1.49%)	5 (0.36%)	16 (1.14%)
Secondary school	100 (7.11%)	35 (2.49%)	65 (4.62%)
High school or equivalent	367 (26.10%)	202 (14.37%)	165 (11.74%)
Trade/technical/vocational training	277 (19.70%)	133 (9.46%)	144 (10.24%)
BA	459 (32.65%)	209 (14.86%)	250 (17.78%)
graduate	154 (10.95%)	103 (7.33%)	51 (3.63%)
Marital status			
Single	549 (39.05%)	221 (15.72%)	328 (23.33%)
Living together	37 (2.63%)	13 (0.92%)	24 (1.71%)
Married	746 (53.06%)	412 (29.3%)	334 (23.76%)
Widowed	10 (0.71%)	6 (0.43%)	4 (0.28%)
Divorced	58 (4.13%)	46 (3.27%)	12 (0.85%)
Separated	6 (0.43%)	5 (0.36%)	1 (0.07%)
Income			
a lot below-average	367 (26.10%)	169 (12.02%)	198 (14.08%)
a little below average income	398 (28.31%)	146 (10.38%)	252 (17.92%)
average income	311 (22.12%)	135 (9.6%)	176 (12.52%)
a little more than average	178 (12.66%)	122 (8.68%)	56 (3.98%)
significantly higher than average	69 (4.91%)	48 (3.41%)	21 (1.49%)
missing data	83 (5.90%)	83 (5.9%)	0

**Table 2:** variance, covariance, and correlations of indicators, Cronbach’s α for constructed latent variable (Israel).

Latent Variable		Indicator	M	SD	1	2	3	4	5	6	7	8	9	10	11
**Complacency**	1	Susceptibility 1	3.09	1.088	**1.184**	*0.534*	*0.396*	*0.435*	*−0.113*	*−0.044*	*−0.022*	*−0.056*	*0.034*	*0.034*	*0.050*
Cronbach’s α = 0.771	2	Susceptibility 2	2.92	1.141	0.430 **	**1.302**	*0.520*	*0.764*	*0.068*	*0.110*	*0.036*	*−0.064*	*−0.003*	*0.034*	*0.051*
	3	Seriousness 1	3.48	1.071	0.340 **	0.425 **	**1.147**	*0.767*	*−0.017*	*−0.001*	*−0.008*	*−0.012*	*0.023*	*0.035*	*0.027*
	4	Seriousness 2	2.88	1.163	0.343 **	0.576 **	0.616 **	**1.354**	*0.145*	*0.209*	*0.069*	*−0.013*	*−0.052*	*0.010*	*0.031*
**constraint**	5	Outcome expectation 1	1.92	0.946	−0.110 **	0.063	−0.017	0.132 **	**0.895**	*0.512*	*0.325*	*−0.025*	*−0.036*	*−0.016*	*−0.052*
Cronbach’s α = 0.543	6	Outcome expectation 2	1.97	0.926	−0.044	0.104 **	−0.001	0.194 **	0.585 **	**0.857**	*0.341*	*0.017*	*−0.046*	*−0.007*	*−0.027*
	7	Efficacy expectation 1	2.16	0.879	−0.023	0.036	−0.008	0.068	0.391 **	0.419**	**0.773**	*−0.034*	*−0.086*	*−0.072*	*−0.088*
	8	Efficacy expectation 2	2.16	0.885	−0.059	−0.064	−0.013	−0.012	−0.030	0.021	−0.044	**0.783**	*−0.016*	*−0.016*	*−0.021*
**confidence**	9	Confidence in government	2.67	0.813	0.038	−0.003	0.026	−0.055	−0.047	−0.062	−0.121 **	−0.022	**0.661**	*0.430*	*0.362*
Cronbach’s α = 0.851	10	Confidence in hospitals	2.55	0.802	0.039	0.037	0.041	0.011	−0.021	−0.009	−0.103 **	−0.022	0.660 **	**0.644**	*0.472*
	11	Confidence in medical workers	2.77	0.791	0.058	0.056	0.031	0.034	−0.070	−0.036	−0.126 **	−0.030	0.563 **	0.744 **	**0.626**

note: ** *p* < 0.001, correlation in lower left, variances along diagonal in bold, and covariances in upper right italicized.

**Table 3:** variance, covariance, and correlations of indicators, Cronbach’s α for constructed latent variable (China).

Latent Variable		Indicator	M	SD	1	2	3	4	5	6	7	8	9	10	11
**Complacency**	1	Susceptibility 1	3.72	1.167	**1.363**	*0.615*	*0.590*	*0.212*	*−0.057*	*−0.003*	*−0.079*	*−0.059*	*0.003*	*0.029*	*0.017*
Cronbach’s α = 0.682	2	Susceptibility 2	3.43	1.104	0.477 **	**1.220**	*0.534*	*0.410*	*−0.017*	*0.025*	*−0.093*	*−0.025*	*0.014*	*0.013*	*0.002*
	3	Seriousness 1	2.60	1.275	0.397 **	0.379 **	**1.625**	*0.508*	*0.118*	*0.109*	*−0.051*	*0.034*	*−0.008*	*−0.020*	*−0.031*
	4	Seriousness 2	2.16	1.125	0.161 **	0.330 **	0.354 **	**1.265**	*0.178*	*0.351*	*0.011*	*0.119*	*−0.020*	*−0.034*	*−0.020*
**Constraint**	5	Outcome expectation 1	1.62	0.822	−0.059	−0.019	0.113 **	0.192 **	**0.675**	*0.282*	*0.211*	*0.259*	*−0.078*	*−0.116*	*−0.068*
Cronbach’s α = 0.707	6	Outcome expectation 2	1.72	0.830	−0.003	0.027	0.103 **	0.376 **	0.413 **	**0.689**	*0.233*	*0.292*	*−0.088*	*−0.098*	*−0.079*
	7	Efficacy expectation 1	1.88	0.912	−0.074 *	−0.093 *	−0.044	0.011	0.281 **	0.308 **	**0.832**	*0.397*	*−0.093*	*−0.130*	*−0.110*
	8	Efficacy expectation 2	1.84	0.877	−0.058	−0.026	0.031	0.120 **	0.360 **	0.401 **	0.496 **	**0.769**	*−0.081*	*−0.116*	*−0.087*
**Confidence**	9	Confidence in government	3.71	0.483	0.005	0.026	−0.013	−0.036	−0.197 **	−0.219 **	−0.210 **	−0.192 **	**0.234**	*0.190*	*0.171*
Cronbach’s α = 0.885	10	Confidence in hospitals	3.62	0.562	0.045	0.021	−0.029	−0.053	−0.251 **	−0.209 **	−0.253 **	−0.236 **	0.701 **	**0.315**	*0.218*
	11	Confidence in medical workers	3.69	0.505	0.028	0.004	−0.049	−0.035	−0.164 **	−0.189 **	−0.239 **	−0.196 **	0.699 **	0.767 **	**0.255**

note: * *p* < 0.05, ** *p* < 0.001, correlation in lower left, variances along diagonal in bold, and covariances in upper right italicized.

**Table 4:** variance, covariance, and correlations of indicators, Cronbach’s α for constructed latent variable (Both).

Latent Variable		Indicator	M	SD	1	2	3	4	5	6	7	8	9	10	11
**Complacency**	1	Susceptibility 1	3.40	1.170	**1.370**	*0.653*	*0.356*	*0.211*	*−0.131*	*−0.063*	*−0.094*	*−0.109*	*0.181*	*0.199*	*0.177*
Cronbach’s α = 0.675	2	Susceptibility 2	3.18	1.151	0.485 **	**1.324**	*0.415*	*0.496*	*−0.012*	*0.036*	*−0.064*	*−0.086*	*0.138*	*0.159*	*0.143*
	3	Seriousness 1	3.04	1.256	0.242 **	0.287 **	**1.577**	*0.794*	*0.116*	*0.109*	*0.032*	*0.083*	*−0.222*	*−0.227*	*−0.204*
	4	Seriousness 2	2.52	1.199	0.150 **	0.359 **	0.528 **	**1.437**	*0.214*	*0.325*	*0.090*	*0.111*	*−0.223*	*−0.203*	*−0.159*
**Constraint**	5	Outcome expectation 1	1.77	0.898	−0.125 **	−0.012	0.103 **	0.199 **	**0.806**	*0.415*	*0.289*	*0.141*	*−0.135*	*−0.145*	*−0.129*
Cronbach’s α = 0.65	6	Outcome expectation 2	1.85	0.888	−0.060 *	0.035	0.098 **	0.305 **	0.521 **	**0.788**	*0.304*	*0.175*	*−0.133*	*−0.120*	*−0.111*
	7	Efficacy expectation 1	2.02	0.907	−0.089 **	−0.061 *	0.028	0.083 **	0.354 **	0.378 **	**0.822**	*0.204*	*−0.163*	*−0.176*	*−0.163*
	8	Efficacy expectation 2	2.00	0.896	−0.104 **	−0.083 **	0.073 **	0.104 **	0.176 **	0.220 **	0.252 **	**0.802**	*−0.134*	*−0.153*	*−0.129*
**Confidence**	9	Confidence in government	3.19	0.848	0.182 **	0.141 **	−0.208 **	−0.220 **	−0.177 **	−0.176 **	−0.211 **	−0.176 **	**0.720**	*0.589*	*0.506*
Cronbach’s α = 0.918	10	Confidence in hospitals	3.09	0.874	0.194 **	0.158 **	−0.207 **	−0.194 **	−0.185 **	−0.154 **	−0.222 **	−0.195 **	0.794 **	**0.765**	*0.590*
	11	Confidence in medical workers	3.23	0.807	0.187 **	0.154 **	−0.201 **	−0.165 **	−0.177 **	−0.155 **	−0.223 **	−0.178 **	0.739 **	0.836 **	**0.651**

note: * *p* < 0.05, ** *p* < 0.001, correlation in lower left, variances along diagonal in bold, and covariances in upper right italicized.

**Table 5:** descriptive statistics for avoidant behaviors.

No	Avoidant Behavior	Israel		China	
		M	SD	M	SD
1	Avoid arriving in places where it is known that the virus is locally transmitted	4.43	1.04	4.65	0.80
2	Do not share your towel with others	3.84	1.35	4.63	0.79
3	Maintain at least 1 metre distance between yourself and anyone who is coughing or sneezing.	4.08	1.03	4.21	0.96
4	Avoid shaking hands	4.49	0.87	4.41	0.81
5	Avoid using common utensils in the same meal.	4.24	1.10	4.33	1.01
6	Do not visit your family members and friends who do not live with you.	4.31	0.88	4.35	0.83
7	Do not going into crowded or airtight places	4.56	0.74	4.39	0.82
8	Put on facial mask when you go outside	2.57	1.50	4.69	0.73
9	Do not use public transportation	4.33	1.12	3.93	1.15

Cronbach’s α = 0.729 for both countries combined, Cronbach’s α = 0.764 for Israel, Cronbach’s α = 0.746 for China.

**Table 6:** 3C-avoidant behavior model.

	Original Model		Multiple-Group Analysis	
Model-Fix Goodness	Israel	China	Israel	China
model fit				
χ^2^	646.396	628.974	1256.452	
df	165	166	333	
χ^2^/df	3.92	3.79	3.773	
RMSEA	0.06	0.06	0.045	
GFI	0.91	0.91	0.912	
SRMR	0.06	0.06	0.0586	
Standardized coefficient				
confidence->constraints	−0.08	−0.400 *	−0.072	−0.400 *
complacency->constraints	0.159 *	0.022	0.154 *	0.022
confidence->avoidant behavior	0.127 *	0.089	0.127 *	0.089
complacency->avoidant behavior	−0.226 *	0.214 *	−0.227 *	0.214 *
constraint->avoidant behavior	−0.260 *	−0.228 *	−0.274 *	−0.228 *

* *p* < 0.05.
